# Metabolomic profiling of triple negative breast cancer cells suggests that valproic acid can enhance the anticancer effect of cisplatin

**DOI:** 10.3389/fcell.2022.1014798

**Published:** 2022-12-05

**Authors:** Avital Granit, Kumudesh Mishra, Dinorah Barasch, Tamar Peretz-Yablonsky, Sara Eyal, Or Kakhlon

**Affiliations:** ^1^ Sharett Institute of Oncology, Hadassah-Hebrew University Medical Center, Jerusalem, Israel; ^2^ Institute for Drug Research School of Pharmacy, The Hebrew University of Jerusalem, Jerusalem, Israel; ^3^ Department of Neurology, Hadassah-Hebrew University Medical Center, Jerusalem, Israel; ^4^ Faculty of Medicine, Hebrew University of Jerusalem, Jerusalem, Israel; ^5^ Mass Spectrometry Unit, Institute for Drug Research, School of Pharmacy, The Hebrew University of Jerusalem, Jerusalem, Israel; ^6^ The Dame Susan Garth Chair of Cancer Research, The David R. Bloom Centre for Pharmacy and Dr. Adolf and Klara Brettler Centre for Research in Molecular Pharmacology and Therapeutics at The Hebrew University of Jerusalem, Jerusalem, Israel

**Keywords:** valproic acid, metabolomics, metabolism, cisplalin, triple negative breast cancer

## Abstract

Cisplatin is an effective chemotherapeutic agent for treating triple negative breast cancer (TNBC). Nevertheless, cisplatin-resistance might develop during the course of treatment, allegedly by metabolic reprograming, which might influence epigenetic regulation. We hypothesized that the histone deacetylase inhibitor (HDACi) valproic acid (VPA) can counter the cisplatin-induced metabolic changes leading to its resistance. We performed targeted metabolomic and real time PCR analyses on MDA-MB-231 TNBC cells treated with cisplatin, VPA or their combination. 22 (88%) out of the 25 metabolites most significantly modified by the treatments, were acylcarnitines (AC) and three (12%) were phosphatidylcholines (PCs). The most discernible effects were up-modulation of AC by cisplatin and, contrarily, their down-modulation by VPA, which was partial in the VPA-cisplatin combination. Furthermore, the VPA-cisplatin combination increased PCs, sphingomyelins (SM) and hexose levels, as compared to the other treatments. These changes predicted modulation of different metabolic pathways, notably fatty acid degradation, by VPA. Lastly, we also show that the VPA-cisplatin combination increased mRNA levels of the fatty acid oxidation (FAO) promoting enzymes acyl-CoA synthetase long chain family member 1 (ACSL1) and decreased mRNA levels of fatty acid synthase (FASN), which is the rate limiting enzyme of long-chain fatty acid synthesis. In conclusion, VPA supplementation altered lipid metabolism, especially fatty acid oxidation and lipid synthesis, in cisplatin-treated MDA-MB-231 TNBC cells. This metabolic reprogramming might reduce cisplatin resistance. This finding may lead to the discovery of new therapeutic targets, which might reduce side effects and counter drug tolerance in TNBC patients.

## Introduction

Breast cancer is the most common cancer and the second death-causing cancer in women worldwide (Siegel et al., 2021). Breast cancer is subdivided into three main molecular subtypes according to the expression pattern of estrogen receptor (ER), progesterone receptor (PR), human epidermal growth factor receptor 2 (HER2), or none of them (triple negative breast cancer, TNBC). TNBC is an aggressive subtype of breast cancer and accounts for 15% of all breast cancer cases (Yin et al., 2020). The main treatment regime for TNBC is chemotherapy. One therapeutic option is cis-diamminedichloroplatinum (II) (cisplatin). Cisplatin was first approved in 1978 for the treatment of bladder and testicular cancer (Prestayko et al., 1979) and has since been used for many different solid malignancies.

The mechanism of action of cisplatin is linked to its ability to crosslink purine bases in the DNA, causing DNA damage and resulting in cell division blockage and apoptotic cell death.

Valproic acid (VPA) is approved as an antiepileptic drug since 1967 (Mattson et al., 1978). VPA possesses an anti-cancer capacity, notably against breast cancer (Wawruszak et al., 2021), which is related to its ability to inhibit histone deacetylase. As histone acetylation enables the interaction of transcription factors and RNA polymerase with DNA, deacetylation of histones and non-histone proteins can modify gene expression and cellular pathways, regulating different functions in cancer cells, such as apoptosis, cell cycle and DNA repair (Brancolini et al., 2022).

Due to the ability of VPA to alter chromatin condensation and transcription and the ability of cisplatin to alkylate DNA, combining both drugs demonstrated a synergistic antitumor activity *in vitro* in different breast cancer cell lines. However, the combination generated a sub-additive (antagonistic) anti-cancer effect when applied in a 1:1 ratio in the TNBC cell line, MDA-MB-231 (Wawruszak et al., 2015). To gain a better understanding of the joint influence of cisplatin and VPA on TNBC cells, we decided to investigate their effect on the intracellular metabolism of MDA-MB-231 cells. To that end, we used targeted metabolomics. Metabolomics is a fast advancing field of research aimed at identifying and quantifying the small molecules (together known as metabolome) involved in attaining metabolic homeostasis (Liesenfeld et al., 2013). Previous metabolomic studies in MDA-MB-231 cells, assessing cisplatin or VPA individually, showed that cisplatin can alter phospholipid biosynthesis (Resendiz-Acevedo et al., 2021) and that VPA can alter the beta-alanine, taurine, and hypotaurine pathways (Zhou et al., 2020). Although these studies demonstrated dramatic metabolic changes in the MDA-MB-231 cells following cisplatin or VPA treatment, there has been no study to date, which compared the metabolomic profile of TNBC cells treated with the VPA-cisplatin combination to cells treated with each drug separately. As HDAC inhibition and cisplatin are key strategies for TNBC therapy, it is important to understand their mutual interaction so as to improve the efficacy of this co-therapy strategy. In the present study, we provide evidence that VPA and cisplatin alone or together can change the levels of carnitine, AC, amino acids (AA), biogenic amines, lipids, and hexose, and the pathways in which they are implicated. Thus our work provides a new perspective on the effect of the VPA and cisplatin combination therapy on TNBC tumors.

## Materials and methods

### Reagents

Sodium valproate was purchased from Merck (KGaA, Darmstadt, Germany). VPA was freshly dissolved in double distilled water (DDW) to a stock concentration of 1 M. Cisplatin was from Pharmachemie B.V. (Haarlem, Netherlands). 3.3 mM stock cisplatin solution in DDW was stored at room temperature. All cell culturing reagents were from Biological Industries Ltd. (Beit HaEmek, Israel). All reagents, internal and calibration standards, quality controls, test mixes, UHPLC column, and a patented 96-well filter plate required for the AbsoluteIDQ®p180 analysis were included in the kit or provided by Biocrates Life Science AG (Innsbruck, Austria). The RNeasy Mini-Isolation Kit was from Qiagen (Hilden, Germany). High-Capacity cDNA Reverse Transcription Kit was from Thermo Fisher scientific (MA, United States). Xpert Fast SYBR was from Grisp (Porto, Portugal). PCR primers used were: CPT1A (forward TCC​AGT​TGG​CTT​ATC​GTG​GTG, reverse CTA​ACG​AGG​GGT​CGA​TCT​TGG); ACSL1 (forward CTT​CTG​GTA​CGC​CAC​GAG​AC, reverse GTC​GCT​GTC​AAG​TAG​TGC​G); FASN (forward CTT​CCG​AGA​TTC​CAT​CCT​ACG​C, reverse TGG​CAG​TCA​GGC​TCA​CAA​ACG); SLC22A5 (forward GAC​CAT​ATC​AGT​GGG​CTA​TTT, reverse CTG​CAT​GAA​GAG​AAG​GAC​AC); SLC25A20 (forward GGG​GTC​ACT​CCC​ATG​TTT​G, reverse TGT​GGT​GAA​TAC​GCC​AGA​TAA​C); and TBP (forward CGG​TTT​GCT​GCG​GTA​ATC, reverse TCT​GGA​CTG​TTC​TTC​ACT​CTT​G).

### Cell lines and cell culture

MDA-MB-231 cells were kindly provided by Prof. Michael Elkin (Hadassah-Hebrew University Medical Center, Jerusalem, Israel) and maintained in Dulbecco’s modified Eagle medium (DMEM) supplemented with 10% fetal calf serum, 1% penicillin, 1% streptomycin, and 1% glutamine. Cells were maintained at 37°C in 5% CO_2_. For treatment, 10^6^ cells were seeded in 10 cm plates, 5 replicate plates per treatment. After 24 h, cells were treated with 10 μM cisplatin alone [the IC50 of cisplatin in MDA-MB-231 cell viability assay was 12 μM (Wawruszak et al., 2015)], with 1 mM VPA [the concentration used for inhibition of HDAC and proliferation in MDA-MB-231 cells (Granit et al., 2018)], or with their combination for 72 h.

### Metabolite extraction

Cells were washed twice with cold saline (0.9% NaCl solution) and detached using a cell scraper. The samples were centrifuged at 1,200 rpm for 3 min at 4°C. The supernatant was removed and ice cold 90% methanol in H_2_O was added to each cell pellet. Three cycles of 3 min sonication at 4°C, snap freeze in liquid nitrogen for 3 min, and thawing were performed. The samples were then centrifuged at 18,000 rpm for 5 min at 4°C. The supernatants were transferred to new tubes and stored at liquid nitrogen until analysis.

### Targeted metabolomics

To capture a broad spectrum of metabolites, we used the AbsoluteIDQ^®^ p180 kit (Biocrates Life Sciences AG, Innsbruck, Austria), targeting 40 AC, 42 AA/biogenic amines, 90 phospholipids, 15 sphingolipids, and hexose, following the manufacturer’s instructions. Briefly, 10 µL of calibration standards, quality controls, and samples were added to the respective wells of the 96-well-based Biocrates sample preparation plate containing a mix of internal standards. After drying the samples under nitrogen, 50 µL of 5% phenyl-isothiocyanate solution were added to each well for derivatization. After incubation for 25 min and subsequent evaporation to dryness under nitrogen, 300 µL of 5 mM ammonium acetate in methanol were added for metabolite extraction, stirred for 30 min and centrifuged. The extracts were diluted with 250 µl of 40% methanol/water. The extracts were analyzed by liquid chromatography with tandem mass spectrometry (LC-MS/MS). This system comprised Nexera UHPLC system (Shimadzu, Kyoto, Japan) coupled to a Triple Quad™ 5500 mass spectrometer (Sciex, Framingham, MA, United States) in electrospray ionization (ESI) mode. AA and biogenic amines were analyzed *via* LC-MS in a positive mode. 5 µL of the sample extract were injected to Biocrates AbsoluteIDQ^®^ p180 kit UHPLC column, 2.1 × 50 mm, protected by a VanGuard^®^ pre-column (Waters, Milford, MA, United States) at 50°C using a 5.8 min solvent gradient employing 0.2% formic acid in water and 0.2% formic acid in acetonitrile. 20 µl of the sample extracts were used in the flow injection analysis (FIA) in the positive mode to capture AC, glycerophospholipids, sphingolipids and hexoses. All FIA injections were carried out using the Biocrates FIA Solvent. All metabolites were identified and quantified using isotopically-labeled internal standards and multiple reaction monitoring (MRM).

### Analysis of mRNA expression

Total RNA was isolated from one million cells using the RNeasy Mini-Isolation Kit according to the manufacturer instructions. The cDNA was synthesized in a 20 μl reverse transcriptase reaction mix containing 1 μg of total RNA. An aliquot of 1 μl reverse-transcribed cDNA was used in each 10 μl PCR reaction, containing Xpert Fast SYBR, and reactions were run on an ABI StepOnePlus PCR system (Thermo Fisher Scientific, MA, United States).

### Data processing and statistical analysis

The LC-MS raw data were quantified using the Analyst 1.6.3 software (Sciex) and exported to the Biocrates MetIDQ™ software. FIA raw data from the AbsoluteIDQ^®^ assay were exported and quantified using the MetIDQ™ software. Quality control samples-based data normalization was performed to minimize the variation of analyses. Initial data cleaning was performed by excluding metabolites with > 20% missing values or values below the limit of detection (LOD) in all experimental groups. Thus, all metabolites with > 80% of the concentration values above the LOD in at least one of the four experimental groups were included for statistical analysis. Remaining missing values were replaced by 1/5 of the minimum positive value of each variable. Data were log-transformed to confirm normal distribution before comprehensive downstream analysis using the web-based tool MetaboAnalyst 5.0 (https://www.metaboanalyst.ca) (Pang et al., 2021). Fold changes (FCs) were calculated to evaluate differences between metabolites in treated samples compared to control samples. FCs >1.5 were considered significant. To correct for multiple comparisons and thus to minimize false positives, false discovery rates (FDRs) were calculated based on the Benjamini–Hochberg procedure (Benjamini and Hochberg, 1995). FDR-corrected *p*-values < 0.05 were considered statistically significant. Both unsupervised principal component analysis (PCA) and supervised partial least squares-discriminant analysis PLS-DA were performed whenever necessary to determine the metabolic signature contributing to group separation. PLS-DA decreases intergroup variability and improves separation. However, PLS-DA is prone to data overfitting. Thus, the quality of the model was assessed by cross-validation (calculation of Q2, R2, and accuracy values) and the overfitting tendency of the model, or the significance of class separations, was tested using permutations. The PLS-DA Variable Importance in Projection (VIP-score) was calculated and metabolites with a VIP score >1 were considered important for group separation. Thus, for Metabolomic Pathway Analysis (MetPA) we only considered metabolites that overlapped between the two different statistical approaches (FDR-corrected *p*-value < 0.05 and VIP score >1 or FDR-corrected *p*-value < 0.05 and FC > 1.5). The Homo sapiens KEGG pathway libraries were used as references for the MetPA. Heatmaps were created using MetaboAnalyst 5.0.

For analysis of mRNA expression and metabolites subgroups concentrations, the Kruskal-Wallis test followed by Dunn’s post-hoc test (Prism ver. 9; Graph Pad, La Jolla, CA, United States (were used to determine the statistical significance of the differences between experimental groups. Data are presented as mean and standard deviation. A *p*-value <0.05 was considered significant.

### Cell viability

We determined the extent of metabolically viable cells by the overall cellular ATP levels using the CellTiter Glo kit (Promega, Madison, WI, United States) according to manufacturer instructions.

### NAD^+^/NADH

NAD^+^/NADH ratio was determined by Promega’s NAD/NADH-Glo kit according to manufacturer instructions.

## Results

### Hierarchical clustering, principal component and VIP analyses

**FIGURE 1:**
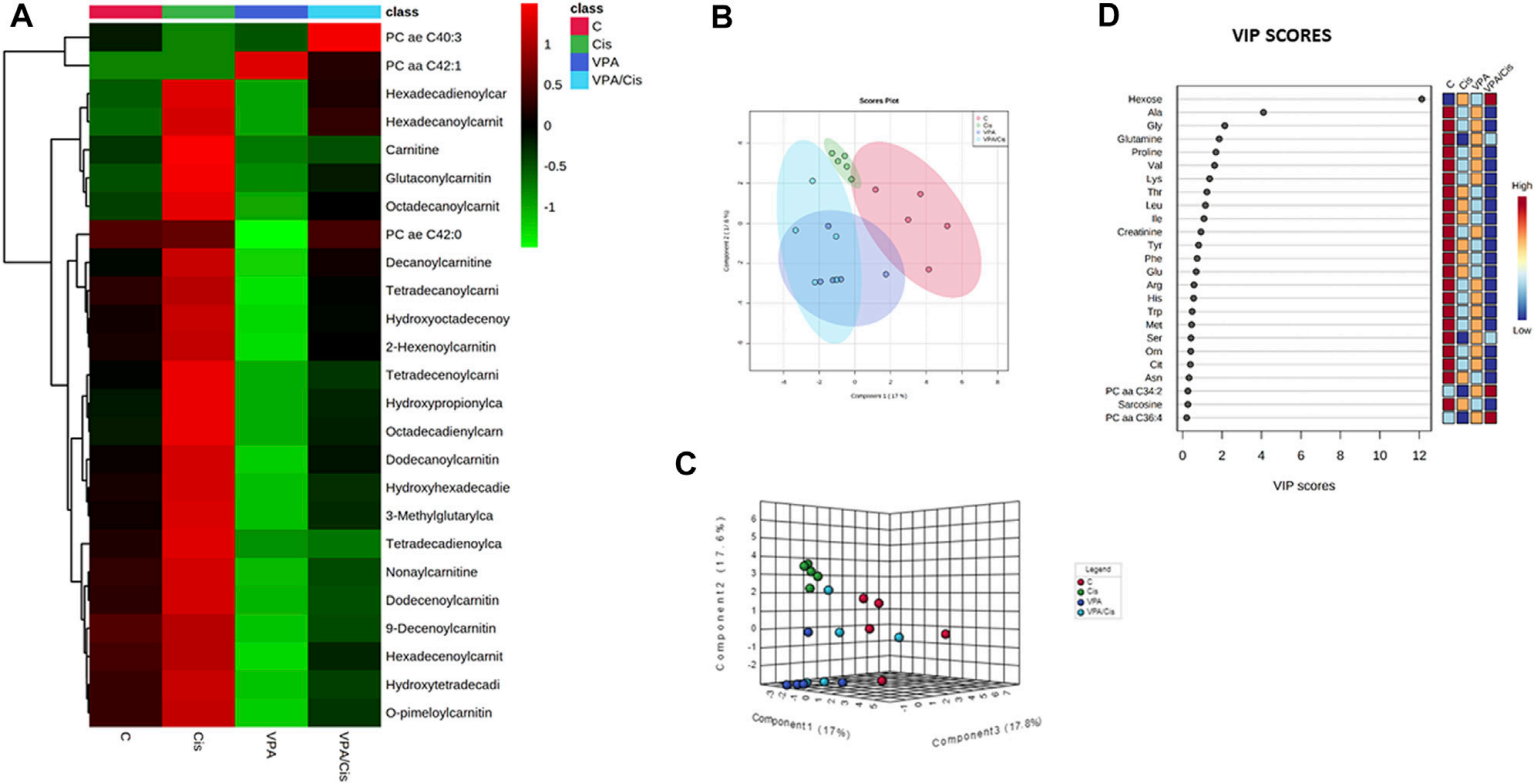
Metabolic analysis of VPA, Cisplatin, their combination and control (untreated) MDA-MB-231 cells after 72 h of treatment. **(A)** Heatmap showing the 25 metabolites most influenced by the treatments. **(B)** PLS-DA supervised score plot. **(C)** 3D supervised PLS-DA score plot. **(D)** VIP score plot. From each condition, n = 5 replicates were analyzed.

The hierarchical clustering metabolomic analysis, represented by a heatmap, shows the 25 hits most significantly modified by the treatments when all experimental groups are considered (Figure 1A). Notably, 22 (88%) out of these hits are acylcarnitines (and carnitine itself) and 3 are PCs. Importantly, the most discernible effects were up-modulation of the levels of these AC by cisplatin and, contrarily, their down-modulation by VPA. When cisplatin and VPA were combined, these changes in AC levels were partially abolished. Because only three PCs were significantly modulated by the treatments, a pattern could not be easily detected for PCs. Our partial least squares-discriminant analysis (PLS-DA, Figures 1B,C) suggests that cisplatin and cisplatin-VPA treated cells were most affected by the treatment (their ellipsoid center of gravity was most distant from that of control) and that cisplatin rendered the cells the most homogenous in terms of metabolite variety as compared to the other groups (represented by larger ellipsoids). The PLS-DA VIP score, which ranks metabolites according to their importance for intergroup separation, revealed 25 metabolites with a VIP-score >0. Hexose received VIP score of 12, followed by the AA alanine (VIP = 4), glutamine, and glycine (VIP = 2). The rest of the list included all the AA-related metabolites except aspartate and two biogenic amines [creatinine and sarcosine (Figure 1D)]. These results are in agreement with the often observed high discriminatory power of these metabolites, where even relatively small quantitative changes can have substantial implications on metabolic heterogeneity.

### Metabolite subgroup profiling

**FIGURE 2:**
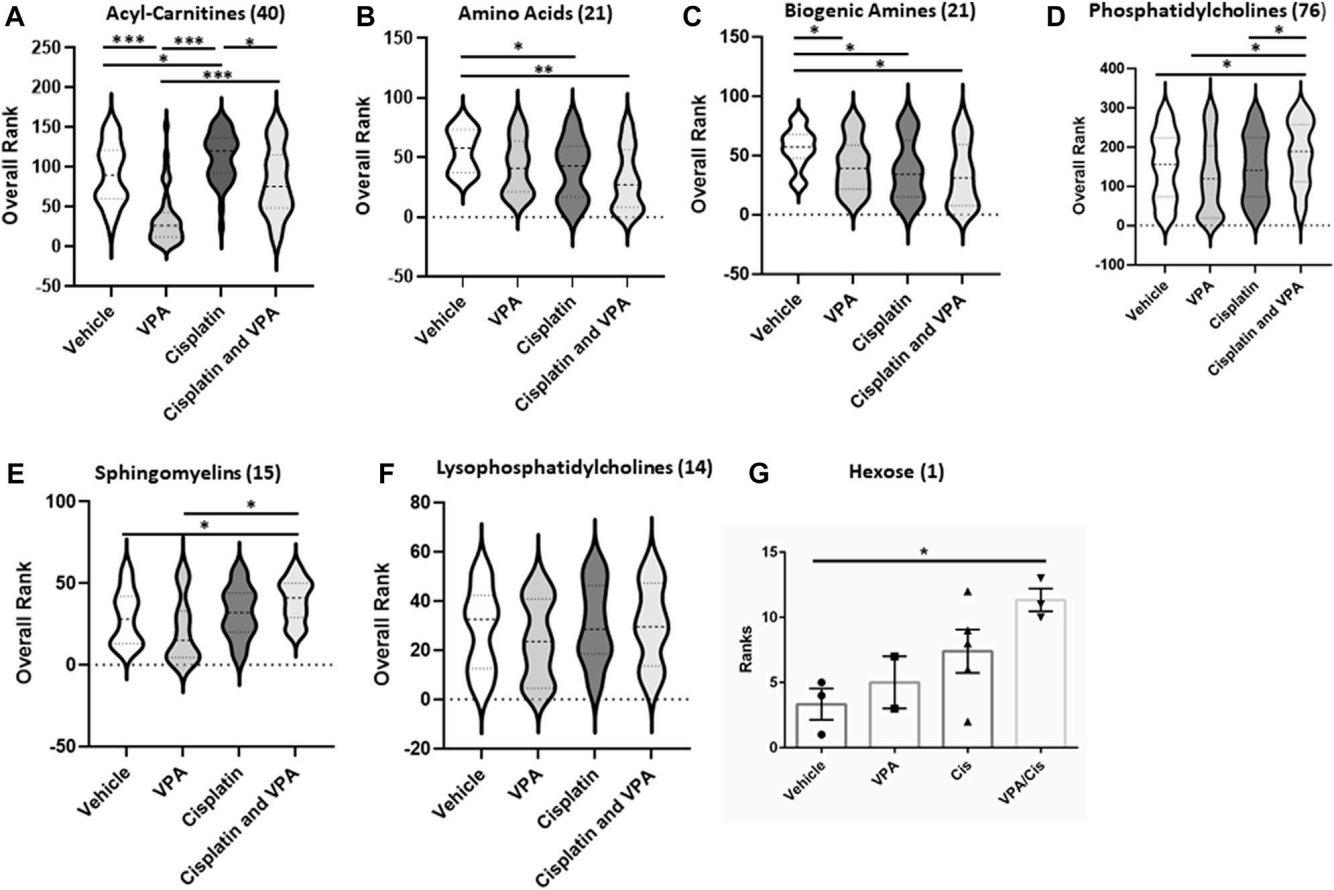
Metabolic subclass analysis of vehicle, VPA, Cisplatin and their combination treated MDA-MB-231 cells after 72 h. Profiling data of overall rank: **(A)** Acyl-Carnitines, **(B)** Amino, **(C)** Biogenic Amides **(D)** Phosphatidylcholines, **(E)** sphingomyelins, **(F)** Lysophosphatidylcholines and **(G)** Hexose; n = 5. The number in the brackets represent the number of metabolites. The middle line in the violin is the mean rank. *p < 0.05, **p < 0.001, ***p < 0.0001.

To further investigate the treatment effects on the metabolome signature, we classified the targeted metabolites analyzed into six groups: AC, AA, biogenic amines, phosphatidylcholines, sphingomyelins, lysophosphatidylcholines and hexose. The concentration rank distribution of the metabolites is displayed as violin plots, where the concentration mean rank is the middle line (Figure 2). As demonstrated for the most affected AC (Figure 1A), VPA also significantly reduced the mean concentration rank of total AC, as compared to vehicle control (*p* < 0.0001), cisplatin (*p* < 0.0001), and the VPA/cisplatin combination (*p* < 0.0001) (Figure 2A, for individual AC, see Supplementary Figure S1A). Cisplatin treatment, on the other hand, increased the AC mean concentration rank as compared to control (*p* < 0.05) and VPA/cisplatin (*p* < 0.05). In the AA group, both cisplatin and VPA/cisplatin treatments reduced the mean concentration rank as compared to control (*p* < 0.03 and *p* < 0.001, respectively, Figure 2B), while VPA also reduced it but only with *p* < 0.06. For two AA, however, glutamate and aspartate, these reductions by cisplatin and VPA/cisplatin were not statistically significant (Supplementary Figure S1B). As opposed to the AC and AA, in the biogenic amines group, all treatments reduced the mean concentration rank as compared to control (*p* < 0.05). The biogenic amines not affected by the treatments were putrescine, spermidine, spermine and taurine (Supplementary Figure S1C). In the lipid groups, mixed trends were observed: While in both the PC and sphingomyelin groups, VPA/cisplatin significantly increased the mean concentration rank over control (*p* < 0.05, Figures 2D,E), no significant difference among treatments was observed in the lysophosphatidylcholines group (Figure 2F). In the hexose group (∼90% D-glucose), the VPA/cisplatin combination was the only treatment which increased (*p* < 0.07) the concentration mean rank (Figure 2G), possibly suggesting glucose accumulation and inhibition of glycolysis by the combination.

**FIGURE 3:**
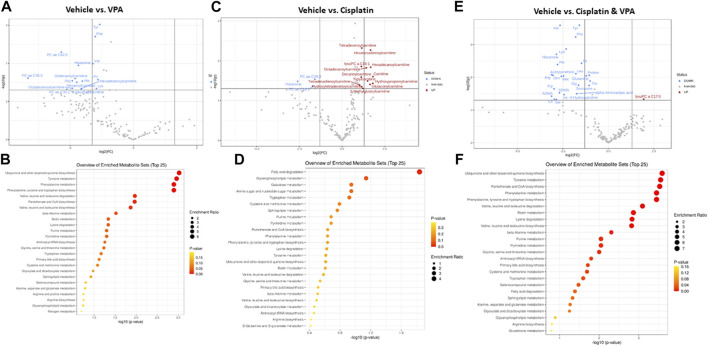
Specific metabolite alterations and metabolic set enrichment analyses (MSEA). Volcano plots with annotated metabolites that were significantly altered in MDA-MB-231 cells following 72 h treatment with **(A)** VPA, **(B)** Cisplatin, **(C)** Cisplatin & VPA, all compared to controls. The red dots represent metabolites above the threshold. The blue dots represent metabolites below the threshold. The further the metabolite’s position away from the (0, 0) coordinate, the more influenced it is by the treatment. Volcano plots show the −log10 FDR-adjusted *p*-values and log2 fold-changes. Metabolites with adjusted *p*-values less than 0.05 and a fold-change greater than 2-fold or less than ½-fold are highlighted and labeled. MSEA show significant metabolic pathway enrichments caused by **(D)** VPA, **(E)** Cisplatin, **(F)** Cisplatin/VPA all compared to controls. The pathways are arranged by *p*-values from pathway enrichment annotation values (X-axis). Node color and radius are based on the *p*-value and enrichment value, respectively.

Specific metabolites and major metabolic pathways modified by the different treatments are presented in volcano plots (Figures 3A,C,E), and metabolite set enrichment analysis (MSEA, Figures 3B,D,F), respectively. Volcano plots highlight the most important [(fold change>2, *p* < 0.05, false discovery rate (FDR) < 0.1] metabolites modified by the different treatments. Metabolite set enrichment analysis (MSEA), on the other hand, was used to demonstrate the effect of these treatments on metabolic pathway enrichment.

VPA (Figure 3A) reduced the abundance of several key AA (tyrosine, phenylalanine, histidine, and lysine), as well as the branched chain AA valine and leucine, all of which are critical for key intracellular biosynthesis and energy transformation pathways. The AC dodecanoylcarnitine, hexadecenoylcarnitine, octadecenoylcarnitine and decenoylcarnitine were also lowered by VPA. In addition, VPA significantly lowered the level of the PCs PC ae C36:3 (PC with acyl-alkyl residue sum C44:5), PC ae C42:0 (PC with acyl-alkyl residue sum C42:0), and PC ae C30:1 (PC with acyl-alkyl residue sum C30:1) and of histamine. The major metabolic pathways influenced by VPA involved ubiquinone biosynthesis, and tyrosine and phenylalanine metabolism and biosynthesis (Figure 3B). The main effect of cisplatin treatment, on the other hand, was the notable increase of 12 different AC (including free carnitine) suggesting inhibition of β-oxidation. Two PCs and histamine were also inhibited by cisplatin (Figure 3C). In agreement with this exclusive effect on AC, cisplatin also exclusively affected the fatty acid degradation pathway (Figure 3D). While significantly increasing a single metabolite, lysoPC C17:0, the combined VPA/cisplatin treatment mainly decreased the levels of different AA (more than VPA alone (Figure 3E, cf Figure 3A) and biogenic amines, such as asymmetric dimethylarginine ADMA. Our MSEA analysis demonstrates that, in contradistinction to cisplatin, the same main metabolic pathways, involving ubiquinone, tyrosine, and phenylalanine, were affected by both VPA and the VPA/cisplatin combination (Figure 3F), suggesting a stronger effect of VPA. Another pathway uniquely enriched by VPA/cisplatin is pantothenate and CoA biosynthesis (Figure 3F).

### The effects of the treatments on fatty acid metabolism and cell viability

**FIGURE 4:**
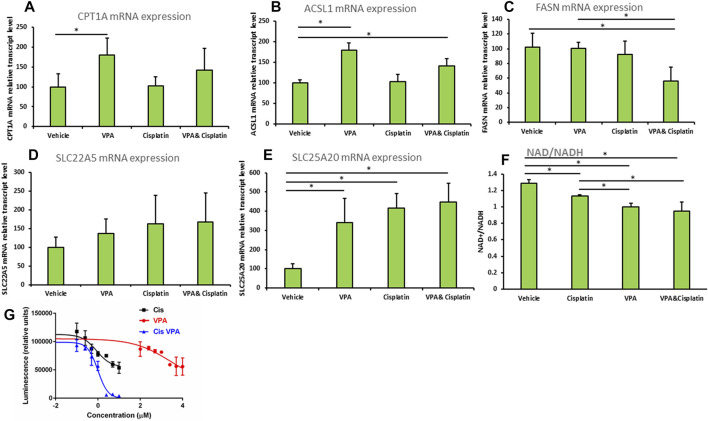
**(A–E)** Expression of beta oxidation genes in MDA-MB-231 cells treated with VPA, cisplatin and their combination. Cells were incubated with 1 mM VPA, 10 µM cisplatin, or their combination for 72 h mRNA expressions of **(A)** CPT1A, **(B)** ASCL1, **(C)** FASN, **(D)** SLC22A5, **(E)** SLC25A20 (by RT-PCR). Results are presented as percent of control (mean ± SD); n = 3 replicates/treatment. **(F)** NAD^+^/NADH was determined in MDA-MB-231 cells treated as indicated. Results shown are means ± SD; n = 3 replicates/treatment. **(G)** ATP-based cell viability was determined in MDA-MB-231 cells treated as indicated. X-axis is presented as log of µg/mL concentration and curves were fitted by a non-linear variable slopes (four parameters) regression. We used 10^(−8) µg/ml instead of the zero concentration to avoid the undefined log (0). Please note that VPA effects on cell viability are comparable to cisplatin’s at molar concentrations which are 2 orders of magnitude higher (Wawruszak et al. (2015) PLoS One 10: e0143013). *, significant difference according to Dunnett’s test, where each treatment was compared to vehicle **(A–E)**, or to Tukey tes, where each treatment was compared with every other treatment **(F)**.

The mRNA expression of carnitine palmitoytransferase 1(CPT1), a rate limiting enzyme in FAO (Qu et al., 2016), was significantly increased 1.8-fold (*p* < 0.01) in cells treated with VPA, as compared to control cells (Figure 4A). The mRNA level of the Acyl-CoA synthetase long chain family member 1 (ASCL1), which esterifies long chain fatty acids into acyl-CoA, further metabolized by FAO (Li et al., 2010), was significantly elevated relative to control by both VPA and VPA/cisplatin treatments (*p* < 0.01 in both, Figure 4B). Carnitine transporters across the plasma membrane (SLC22A5, Figure 4C), or mitochondrial inner membrane (SLC25A20, Figure 4D), were respectively not affected, or equally increased by all treatments. Thus VPA (and to a lesser extent VPA/cisplatin) putatively affected FAO by up-modulation of its committed biochemical steps—acyl-CoA and subsequent acylcarnitine synthesis—and not by affecting carnitine transport. In contrast, the mRNA expression of fatty acid synthase (FASN), committed to synthesis, rather than degradation, of fatty acids was reduced by half in VPA/cisplatin treated cells as compared to control (*p* < 0.01) or VPA treated cells (*p* < 0.01) (Figure 4E).

We predicted that by reducing the FAO precursor carnitine, supplementation of VPA to cisplatin would reduce both FAO and cell viability. We thus indirectly estimated the extent of FAO by the NAD^+^/NADH ratio (Akie et al., 2015), which correlates with OxPhos that is significantly driven by FAO in MDA-MB-231 and most other cell lines. Indeed we show that the order of NAD^+^/NADH-estimated FAO rate is vehicle > cisplatin > VPA > VPA/cisplatin (Figure 4F) and, importantly, that supplementation of VPA to cisplatin significantly decreases FAO. Correspondingly, our ATP-based dose-response cell viability curves show that supplementation of VPA to cisplatin-treated MDA-MB-231 significantly reduces their viability (Figure 4G). Please note that the Area Under the Curve, or bottom of the VPA/cisplatin curve, is significantly lowered as compared to cisplatin alone [from 54777 to −627 (arbitrary units) according to the non-linear regression] and so the insignificant change in IC50 (from 0.262 μg/ml in cisplatin to 0.300 in µg/mL in VPA/cisplatin) cannot be used to estimate the change in viability.

## Discussion

Cisplatin is one of the most efficient anticancer drugs currently used for treating many types of cancer, including TNBC (Tchounwou et al., 2021). However, tumor cells may acquire resistance to cisplatin. One suggested mechanism of resistance is the ability to reprogram the cellular metabolism (Wang et al., 2021), as demonstrated, for instance, by cisplatin resistant gastric cancer cells, which manifested higher rate of glycolysis (Qian et al., 2017). In addition, metabolic reprogramming often involves epigenetic modulation (Sun et al., 2021a). For example, acetyl-CoA is a central metabolite and functions as a carbon source for histone acetylation (Cai et al., 2011). The aim of this work was to compare the metabolomic landscapes of cisplatin, HDACi (VPA) and the HDACi/cisplatin combination in order to gain insight into how metabolic reprogramming by these treatments can modulate therapy resistance and possibly improve it.

Our metabolomic analysis demonstrated that the most distinct metabolic shift caused by the treatments was in AC (Figure 1A and Figure 2A). VPA reduced and cisplatin increased AC levels. On the other hand, the VPA/cisplatin combination reinstated AC to control levels, apparently suggesting that the VPA/cisplatin combination might contribute to therapy resistance by mitigating cisplatin’s anti-cancer effect caused by AC accumulation and inhibition of FAO. However, increasing cisplatin tumor resistance by VPA would require stimulation, rather than inhibition, of FAO. The alleged enhancement of cisplatin’s anticancer effect by VPA (or VPA/cisplatin) can thus be ascribed to their reduction of free carnitine as a FAO precursor (Supplementary Figure S1A). Free carnitine reduction is expected to inhibit, rather than stimulate, FAO and thus VPA is expected to enhance, rather than limit, the anti-cancer effect of cisplatin:

Carnitine is a small, polar molecule *de novo* synthesized from two AA, lysine and methionine. Carnitine facilitates fatty acids transport into the mitochondria to generate FAO substrates for mitochondrial FAO (Reuter and Evans, 2012). Cisplatin inhibits mitochondrial FAO (Li et al., 2020) by inducing oxidative stress, DNA damage and mitochondrial dysfunction (Marullo et al., 2013). In kidney cells, it was discovered that cisplatin can inhibit the plasma membrane carnitine transporter organic cation/carnitine transporter 2 (SLC22A5) (Lancaster et al., 2010), in agreement with the increase in plasma carnitine levels observed in cancer patients upon cisplatin treatment (Heuberger et al., 1998). However, in our hands in TNBC cells, cisplatin increased, rather than decreased, intracellular free carnitine levels (Figure 1A and Figure 3C) with no significant (Figure 4C) or specific (Figure 4D) effects on carnitine transporters. On the other hand, similar to other FAO inhibitors, cisplatin treatment led to AC accumulation. This phenotype of increased free carnitine and AC is atypical of FAO blockade characterized by relatively low free carnitine and increased AC (Semba et al., 2017). Thus we surmise that cisplatin had an intermediate inhibitory effect on FAO. VPA, on the other hand, reduced both free carnitine and AC (Figure 1A and Supplementary Figure S1A), purportedly causing a more pronounced FAO inhibition than cisplatin, which necessitated compensatory up-modulation of the FAO enzymes CPT1 and ASCL1 (Figure 4). The reductions in AC and subsequent FAO are probably attributed to the free carnitine substrate reduction and agree with the documented VPA-mediated FAO inhibition: VPA is a short-chain fatty acid, which requires carnitine for its mitochondrial β-oxidation. VPA combines with carnitine in the mitochondrial inner membrane *via* carnitine acyltransferases (CPT1), acting as the carnitine sink valproylcarnitine, which is eliminated in the urine (Lheureux and Hantson, 2009). However, valproylcarnitine makes only a minor contribution to carnitine sequestration by VPA (Lheureux and Hantson, 2009). VPA also inhibits carnitine biosynthesis by lowering the levels of α-ketoglutarate, a cofactor of the carnitine biosynthesizing enzyme butyrobetaine hydroxylase (Farkas et al., 1996), by restricting restoration of carnitine from AC *via* CPT2 (Lheureux and Hantson, 2009), and by inhibiting SLC22A5-mediated uptake of serum carnitine (Sun et al., 2021b). Limiting the levels of carnitine as an AC substrate resulted in FAO mitochondrial dysfunction (Lheureux and Hantson, 2009; Choi et al., 2015).

It is well established that FAO is the major source for electrons in mitochondrial electron transport chain (ETC) in cancer cells and that fatty acids, rather than glycolysis, are a major source of electrons for ATP production (Lee et al., 2020). The lack of ATP as a substrate for AA synthesis, for instance in the urea cycle, might reduce the levels of AA and biogenic amines in the VPA or cisplatin treatments, and especially in the VPA/cisplatin treatment (Figures 2B,C).

In summary, our main argument is that VPA counters cisplatin tolerance by further inhibiting FAO (Figure 4F), so that when supplemented to cisplatin it can enhance its cancer cell killing capacity (Figure 4G). While cisplatin causes AC accumulation, suggesting lack of degradation of the AC-generated acyl-CoA, it also decreases the levels of carnitine as the ultimate FAO precursor and so its FAO inhibition is limited. VPA, on the other hand, decreases carnitine and also, probably as a consequence, AC, thus further inhibiting FAO and cell viability.

While total biogenic amines were down-modulated by all treatments by a still unknown mechanism (Figure 2C), the biogenic amines putrescine, spermidine, spermine and taurine were not modified by any treatment (Supplementary Figure S1C). Putrescine, spermidine and spermine are cationic molecules that are sequentially synthesized from ornithine and are essential for eukaryotic cell growth and differentiation (Agostinelli et al., 2010). As opposed to our observations, cisplatin has been elsewhere reported to reduce putrescine levels, without changing the level of spermidine and spermine, through modulation of ornithine decarboxylase (ODC) (Geck et al., 2020). These differences might be attributed to the different TNBC cell lines, cisplatin concentration and exposure time used. For VPA, however, it is still not known how it affects putrescine, spermidine and spermine and it is also not known how cisplatin and VPA affect taurine.

Finally, an increase in hexose levels was observed only following treatment with the VPA/cisplatin combination (Figure 2G). This result is also in support of a possible relief of cisplatin resistance brought about by combining cisplatin with VPA. The hexose (90% D-glucose) accumulation caused by the combination suggests inhibition of glycolysis, which could be cytotoxic to TNBC cells.

We also tested the effects of the different treatments on metabolic pathways enrichment. The VPA (Figure 3B) and VPA/cisplatin (Figure 3F) treatments are associated with ubiquinone, tyrosine, and phenylalanine metabolism (Figure 3B). Ubiquinone is an electron-shuttle in the mitochondrial respiratory chain (Lester and Crane, 1959). Ubiquinone’s head group is derived from the essential AA phenylalanine, which is converted into tyrosine (Olson et al., 1963). VPA could interfere with the respiratory chain by inhibiting cytochrome c oxidase (COX), an ETC rate limiting enzyme (Salsaa et al., 2020), FAO, as shown above [Figure 4F and (Silva et al., 2008)], or the TCA cycle (El Hage et al., 2012), thus affecting ubiquinone and its chemical equilibrium. The cellular pathway most affected by cisplatin is the fatty acid degradation pathway (Figure 3D), or FAO as explained above (Figure 4F).

In our study, only VPA increased the mRNA expression of CPT1 and ACSL1 (Figures 4A,B). This finding is in contrast with the observed inhibition of CPT1A by VPA in hepatocytes (Aires et al., 2010) and might subserve a compensatory up-modulation of FAO, which was inhibited by VPA more than cisplatin (Figure 4F). *De novo* synthesis of fatty acids relies on a key rate limiting enzyme, fatty acid synthase (FASN) (Kuhajda, 2006). Many cancer cells depended on FASN for proliferation and survival, which, in turn, are dependent on the synthesis of biological membranes (Currie et al., 2013). According to our results, the VPA/cisplatin combination decreased the mRNA expression of FASN (Figure 4E). The apparent discrepancy between the VPA/cisplatin-mediated increase in PC levels and decrease in FASN mRNA expression suggests negative feedback compensation between the two.

Lastly, our study has some limitations. Firstly, while MDA-MB-231 is commonly accepted as a representative TNBC cell line, it is still only 1 cell line. As this is a proof-of-concept short study, we opted to first determine whether at all cisplatin and VPA can significantly modify the metabolomic landscape before embarking on more comprehensive studies including several cell lines, chemotherapies and HDACi’s. Secondly, we used targeted metabolomics with defined metabolites. Some metabolites were not analyzed in this method, thus additional, possibly untargeted, metabolomic analysis should be performed to obtain a wider view of the metabolomic changes. Thirdly, to confirm our results, it would be interesting to investigate the metabolome in primary cells derived from breast cancer patients receiving cisplatin, VPA or VPA/cisplatin in clinical settings.

In summary, our results, schematically summarized in Supplementary Figure S2, showed that treatment with VPA, cisplatin, or both alters the metabolome of MBA-MB-231 TNBC cells. Especially AC, which are enhanced by cisplatin, were influenced, but also AA, lipids and biogenic amines. Separately, cisplatin and VPA might lead to mitochondrial ETC and FAO dysfunction. However, the VPA/cisplatin combination also presents additional effects, such as further FAO inhibition, and further reductions in AA or biogenic amines levels, by which it could modify cisplatin monotherapy (VPA is often not used as a monotherapy for TNBC) and enhance its anticancer effect. The exact underlying mechanism of these modifications remains to be further explored. Therefore, novel treatments targeting the reprogramed metabolism might diminish the resistance of current therapies and increase the overall survival of TNBC patients.

## Data Availability

The raw data supporting the conclusion of this article will be made available by the authors, without undue reservation.
