# Supplementary material for: Metabolomic profiling of triple negative breast cancer cells suggests that valproic acid can enhance the anticancer effect of cisplatin
Source: Front Cell Dev Biol. 2022 Dec 5;10:1014798. doi: 10.3389/fcell.2022.1014798 (PMC9760697; doi:10.3389/fcell.2022.1014798)
Supplement: Supplementary file 1 [file DataSheet1.docx]

**Figure S1:** Single metabolite analysis of MDA-MB-231 cells treated for 72 h with vehicle, VPA, cisplatin, and VPA/cisplatin. Metabolite concentrations were normalized to the vehicle control. Metabolites analyzed were subdivided to: (A) Acylcarnitines, (B) Amino acids, (C) Biogenic amines, (D) Lysophosphatidylcholines, (E) Phosphatidylcholines, and (F) sphingomyelins. n = 5 replicates. The number in the brackets is the number of metabolites.


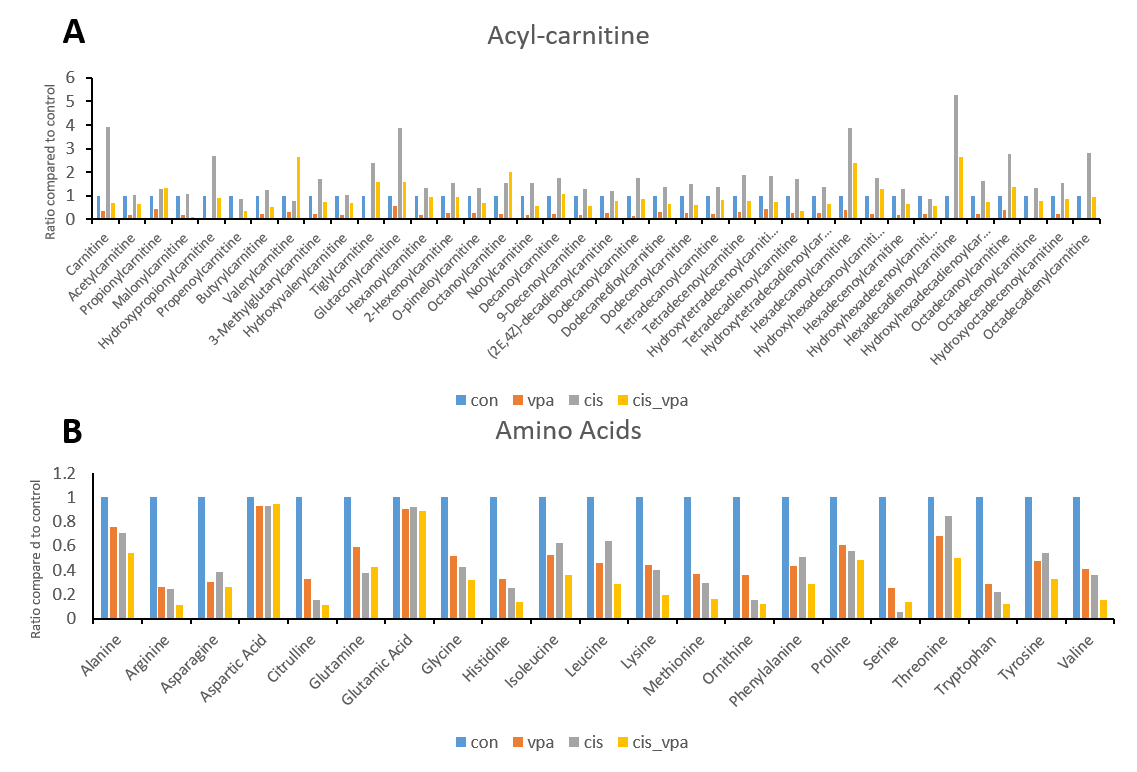


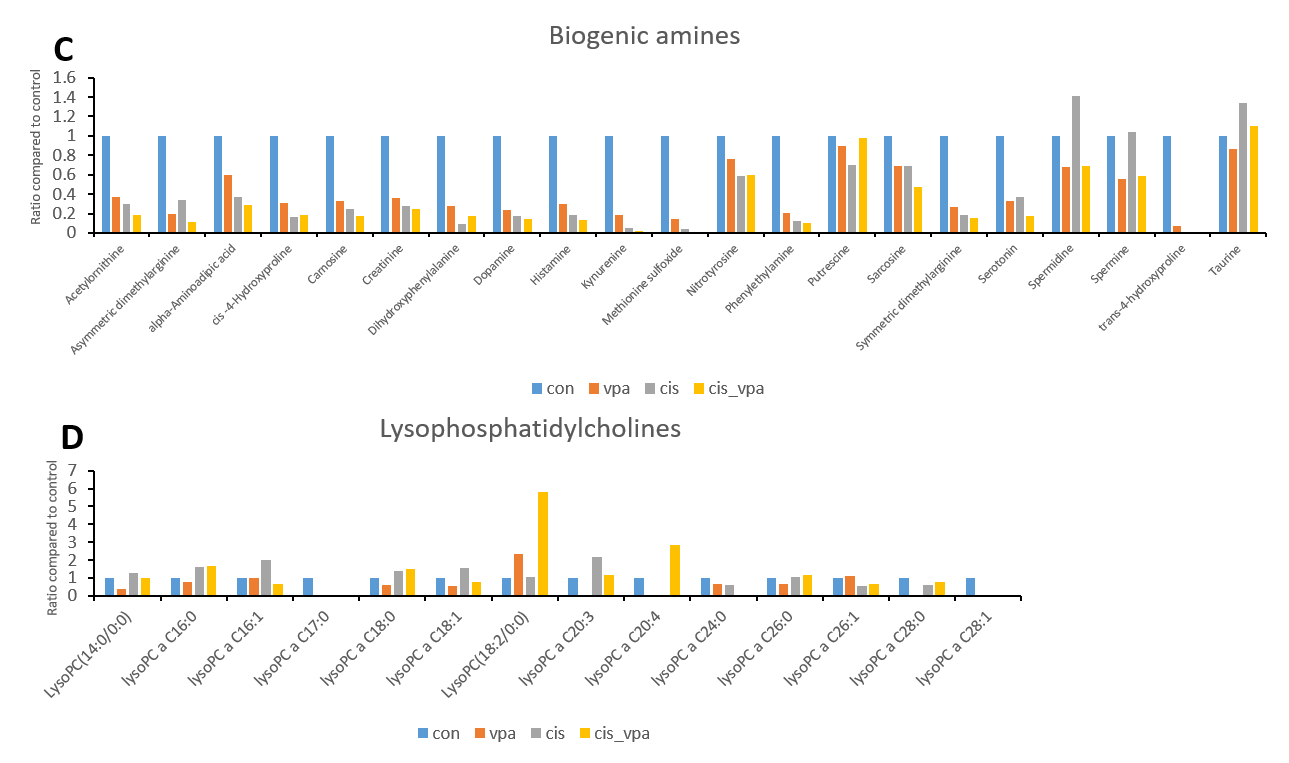


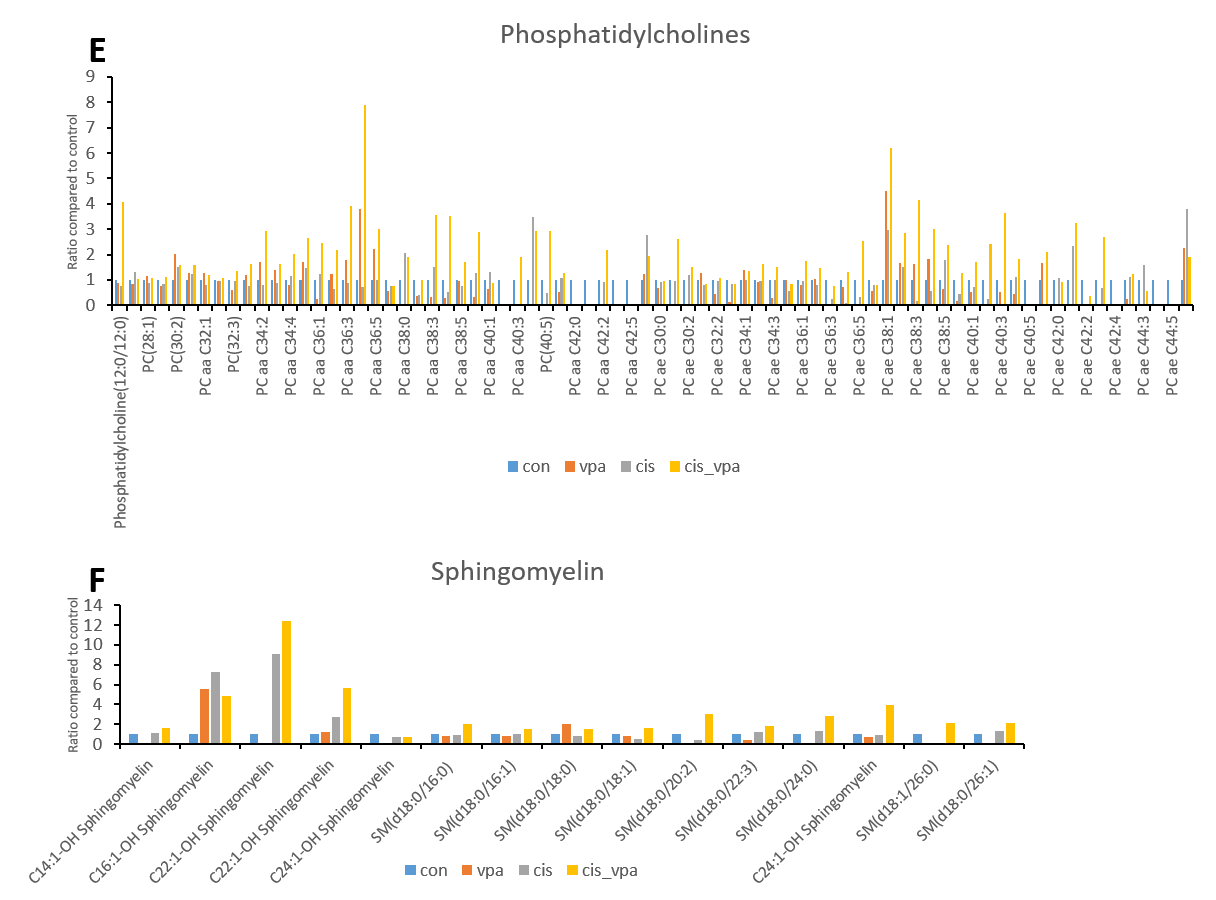


**Figure S2. Summary scheme:** Effects of drugs used on different metabolic groups is shown. Arrows indicate the direction of the effect. Length of arrows (long or short) indicates the extent of the effect. Red arrows show putative enhancers of cancer cell cytotoxicity and/or suggested reversers of cisplatin tolerance in TNBC.

| *Effects compared to control:* | Cisplatin | VPA | VPA/Cisplatin |
| --- | --- | --- | --- |
| Acylcarnitines |  |  |  |
| Free Carnitine |  |  |  |
| Phosphatidylcholines/Sphingomyelins |  |  |  |
| Amino Acids |  |  |  |
| Hexose |  |  |  |
| Biogenic Amines |  |  |  |
| Enzymes of Fatty Acid Oxidation |  |  |  |
